# Signaling Peptides Regulating Abiotic Stress Responses in Plants

**DOI:** 10.3389/fpls.2021.704490

**Published:** 2021-07-19

**Authors:** Jin Sun Kim, Byeong Wook Jeon, Jungmook Kim

**Affiliations:** ^1^Department of Bioenergy Science and Technology, Chonnam National University, Gwangju, South Korea; ^2^Department of Integrative Food, Bioscience and Technology, Chonnam National University, Gwangju, South Korea; ^3^Kumho Life Science Laboratory, Chonnam National University, Gwangju, South Korea

**Keywords:** signaling peptides, receptor-like kinases, dehydration, salt, reactive oxygen species

## Abstract

As sessile organisms, plants are exposed to constantly changing environments that are often stressful for their growth and development. To cope with these stresses, plants have evolved complex and sophisticated stress-responsive signaling pathways regulating the expression of transcription factors and biosynthesis of osmolytes that confer tolerance to plants. Signaling peptides acting like phytohormones control various aspects of plant growth and development via cell-cell communication networks. These peptides are typically recognized by membrane-embedded receptor-like kinases, inducing activation of cellular signaling to control plant growth and development. Recent studies have revealed that several signaling peptides play important roles in plant responses to abiotic stress. In this mini review, we provide recent findings on the roles and signaling pathways of peptides that are involved in coordinating plant responses to abiotic stresses, such as dehydration, high salinity, reactive oxygen species, and heat. We also discuss recent developments in signaling peptides that play a role in plant adaptation responses to nutrient deficiency stress, focusing on nitrogen and phosphate deficiency responses.

## Introduction

As sessile organisms, plants are constantly exposed to a wide range of abiotic stresses, such as drought, high salinity, cold, heat, flooding, and toxic metals in the soil, which negatively affect plant growth, fertility, development, metabolism, photosynthesis, and immune response and impair plant yield and quality in the field (Jeon and Kim, 2013; Suzuki et al., 2014; Ohama et al., 2017; Casal and Balasubramanian, 2019; Hayes et al., 2021). Plants have evolved sophisticated and complex metabolic pathways, signaling modules, such as Ca^2+^-calcineurin B-like (CBL)-CBL-interacting protein kinases (CIPKs) and mitogen-activated protein kinases (MAPKs) modules, and stress-responsive transcription factors for gaining stress tolerance (Qin et al., 2011; De Zelicourt et al., 2016; Zhu, 2016; Shi et al., 2018; Tang et al., 2020). Recent studies have revealed that signaling peptides acting like phytohormones regulate various biochemical, developmental, and physiological processes to coordinate diverse aspects of plant growth and development (Matsubayashi, 2014; Oh et al., 2018; Fletcher, 2020; Jeon et al., 2021; Kim et al., 2021). Signaling peptides are typically small peptides comprising 5–20 amino acids in length or peptides of 40–100 amino acids; they are processed from precursor proteins or directly translated from small open reading frames without proteolytic processing (Matsubayashi, 2014; Oh et al., 2018). These peptides can be mobile in a long or short distance or membrane-bound and are typically recognized by the membrane-localized leucine-rich repeat (LRR)-receptor-like kinases (RLKs), mostly in association with shape-complementary coreceptors. This ligand-receptor/coreceptor association initiates intracellular signaling to control plant growth and development. Several peptides controlling adaptation and tolerance mechanisms in response to abiotic stress have been identified. In this review, we summarize the roles and signaling pathways of small peptides involved in coordinating plant responses against abiotic stresses, such as dehydration, high salinity, reactive oxygen species (ROS), and heat, and discuss future directions on the peptide research in abiotic stress response. The roles of signaling peptides regulating plant responses under nitrogen or phosphate deficiency are also discussed.

## Dehydration Stress Response

### CLE Peptides

**FIGURE 1:**
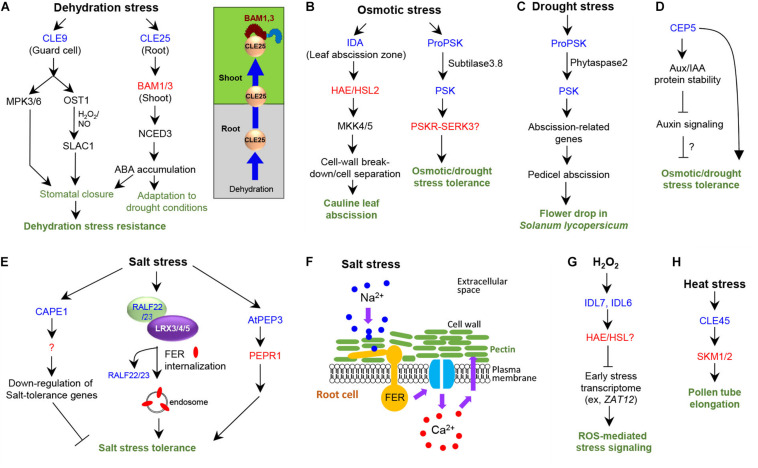
Signaling peptides control abiotic stress responses in plants. Blue and red letters indicate peptides and the cognate receptors, respectively. Plant not specified in the subtitle indicates *Arabidopsis*. **(A)** Roles and signaling pathways of CLE9/25 in dehydration stress response. **(B)** Roles and signaling pathways of IDA and PSK in osmotic stress response. **(C)** Roles of PSK in flower abscission in tomato under drought stress. **(D)** Role of CEP5 in osmotic and drought stress tolerance. **(E)** Roles and signaling pathways in high salinity stress response. **(F)** Role of FER receptor kinase during high salinity stress response to maintain cell-wall integrity in root cells. **(G)** Role of IDL6/7 in ROS-mediated stress signaling. **(H)** Role of CLE45 in pollen tube elongation under heat stress.

The CLAVATA3(CLV)/EMBRYO-SURROUNDING REGION-RELATED (CLE) peptides are a major group of signaling peptides in plants (Goad et al., 2017), usually 12–14 amino acids long and processed from a large precursor protein (Strabala et al., 2014). Several CLE peptides play important roles in root meristem maintenance, vasculature tissue and shoot development, and stomata formation and function (Oh et al., 2018; Fletcher, 2020; Jeon et al., 2021). In particular, *CLE25* and *CLE9* mediate dehydration stress tolerance response in *Arabidopsis thaliana* (Arabidopsis) (Takahashi et al., 2018; Zhang et al., 2019; Figure 1A).

*CLE25* is a mobile peptide that links water deficit to abscisic acid (ABA)-mediated tolerance to dehydration stress (Takahashi et al., 2018; Figure 1A). Water deficiency upregulates *CLE25* expression in root vasculature; peptides then migrate to the leaves, inducing stomatal closure (Takahashi et al., 2018; Figure 1A). *CLE25* peptides applied to the roots can induce expression of the gene encoding the enzyme NINE-CIS-EPOXYCAROTENOID DIOXYGENASE3 (NCED3), which cleaves an ABA precursor molecule and generates bioactive ABA (Figure 1A). The *cle25* knockout mutants are more sensitive to dehydration than wild type (Takahashi et al., 2018). Grafting assays showed that root-derived *CLE25* during dehydration stress is recognized by BARELY ANY MERISTEM1 (BAM1) and BAM2 receptors only in the leaves but not in the roots (Takahashi et al., 2018); the *bam1 bam2* mutants are dehydration-sensitive (Takahashi et al., 2018). These results suggest that *CLE25*–BAM modules convey dehydration signals in the soil from the roots to the leaves in a long-distance signaling to induce ABA accumulation via upregulating *NCED3* expression for stress adaptation and resistance against drought conditions.

*CLE9* plays a role in enhancing drought tolerance by regulating stomatal closure (Zhang et al., 2019; Figure 1A). Exogenous application of *CLE9* peptides or overexpression of *CLE9* induces stomatal closure and enhances drought tolerance. Genetic analysis showed that *CLE9*-induced stomatal closure requires MITOGEN-ACTIVATED PROTEIN KINASE (MAPK)3 (MPK3)/6 and two guard cell ABA-signaling components–protein kinase OPEN STOMATA 1 (OST1) and anion channel protein SLOW ANION CHANNEL-ASSOCIATED 1 (SLAC1) (Zhang et al., 2019; Figure 1A). Moreover, the production of hydrogen peroxide and nitric oxide is stimulated by *CLE9* and disappears in NADPH oxidase-deficient or nitric reductase mutants, respectively (Zhang et al., 2019). These findings indicate that *CLE9* controls stomatal closure in response to drought stress by sharing OST1 and SLAC1, thereby enhancing drought stress tolerance. *CLE9* is specifically expressed in guard cells (Zhang et al., 2019); hence, *CLE9*, unlike *CLE25*, acts locally to control stomatal closure. Further, the *bam*/*clv1* mutants respond normally to *CLE9* peptides in stomatal closure, indicating that BAM receptors are unlikely to be involved in the recognition of *CLE9* peptides (Zhang et al., 2019).

### IDA Peptide

The INFLORESCENCE DEFICIENT IN ABSCISSION (IDA) peptide functions in floral organ abscission and lateral root emergence through the LRR-RLKs, HAESA (HAE), and HAESA-LIKE 2 (HSL2) receptors and the MAPK KINASE4 (MKK4)/5-MPK3/6 cascade (Butenko et al., 2003; Stenvik et al., 2006; Cho et al., 2008; Kumpf et al., 2013; Zhu et al., 2019). The extended 20-amino acid Pro-rich motif at the C terminus of IDA, was sufficient to induce floral abscission in Arabidopsis (Stenvik et al., 2008). A 12-amino acid peptide with hydroxylation of a Pro residue at position 7 was identified as the most efficient peptide for signaling activation through HSL2 receptor in *Nicotiana benthamiana* leaf tissue (Butenko et al., 2014). *IDA* is involved in drought-induced cauline leaf abscission (Patharkar and Walker, 2016). Expression of *HAE* and *IDA* is induced in leaf abscission zones when the leaves are subject to drought conditions (Patharkar and Walker, 2016). Mutant analysis showed that *IDA*, *HAE*/*HSL2*, and *MKK4*/*5* are all necessary for drought-induced cauline leaf abscission (Figure 1B) as they function in floral organ abscission and lateral root emergence (Patharkar and Walker, 2016). *IDA* induces cell-wall breakdown and thus cell separation, causing these organ abscission events (Stenvik et al., 2006; Zhu et al., 2019; Figure 1B).

### PSK Peptide

Phytosulfokine-α (PSK-α) is a pentapeptide sulfated at two Tyr residues (Stührwohldt et al., 2011). Seven Arabidopsis loci encode putative *PSK* precursor genes (Kaufmann and Sauter, 2019). PSK-PSK receptor (PSKR)/coreceptor SOMATIC EMBRYOGENESIS RECEPTOR KINASE 3 (SERK3) modules promote cell elongation and primary/lateral root growth (Amano et al., 2007; Ladwig et al., 2015; Wang et al., 2015). PSK and subtilases (SBTs), which cleave PSK precursor protein for generating the PSK pentapeptide, play a role in drought stress tolerance in Arabidopsis (Stührwohldt et al., 2021). Four *PSK* genes–*PSK1*, *PSK3*, *PSK4*, and *PSK5*–and three *SBT* genes*–SBT1.4*, *SBT3.7*, and *SBT3.8–*are significantly upregulated in response to osmotic stress, such as mannitol treatment (Stührwohldt et al., 2021). Shoot and root growth is slower in *sbt3.8* mutant plants than in wild type under osmotic stress, whereas the osmotic stress-induced sensitive phenotype in *sbt3.8* can be recovered by PSK peptide treatment. Arabidopsis plants overexpressing the PSK precursor (*proPSK1*) exhibit enhanced root and hypocotyl growth and osmotic stress tolerance. ProPSK1 is cleaved by SBT3.8 at the C-terminus of the PSK peptide. ProPSK1 processing depends on the Asp residue directly following the cleavage site and is impaired in the *sbt3.8* mutant. Moreover, *SBT3.8* overexpression in Arabidopsis improves osmotic stress tolerance and induces shoot and root growth. These results suggest that SBT3.8 mediates PSK peptide processing from the precursor protein proPSK1, thus contributing to drought stress tolerance (Figure 1B).

Phytosulfokine signaling controls drought-induced flower drop in tomato (Reichardt et al., 2020). Overexpression of phytaspase 2 (phyt2), a subtilisin-like protease that cleaves proPSK protein, in tomato plants (*Solanum lycopersicum*) enhances premature flower abscission, whereas *SlPhyt2* knockdown reduces flower drop under drought stress conditions, implying a role of *SlPhyt2* in drought-induced abortion of flower and fruit development (Reichardt et al., 2020). Consistently, *SlPhyt2* expression is induced in response to drought stress in flower pedicels proximal to the abscission zone. Expression of *SlPSK1* and *SlPSK6* is coinduced with that of *SlPhyt2* by drought stress. Using a proteomics assay with a substrate library, the PSK precursor was identified as a candidate substrate for SlPhyt2. Peptides harboring the PSK pentapeptide of varying sizes can be cleaved by SlPhyt2 in an Asp-specific manner, releasing mature PSK *in vitro*. Mature PSK induced pedicel abscission in an inflorescence bioassay. PSK treatment upregulated tomato abscission-related polygalacturonase genes and downregulated genes that maintain the abscission zone in an inactive state, indicating that PSK acts as a signal for pedicel abscission in tomato. These findings suggest that the subtilase SlPhyt2, expressed in the pedicel, produces bioactive PSK, which then triggers abscission by inducing cell wall hydrolases in the abscission zone in response to drought stress (Figure 1C).

### CEP5 Peptide

The *C-terminally encoded peptide* (*CEP*) genes encode proteins comprising an N-terminal secretion signal, a variable domain, one or more CEP domains, and a short C-terminal extension (Ogilvie et al., 2014). The CEP precursor proteins undergo proteolysis and Pro hydroxylation to become a bioactive 15-amino acid CEP peptide (Ohyama et al., 2008). CEPs and the CEP receptors, CEPR1/2, play diverse roles in plant responses to changing environmental conditions, such as the systemic N-acquisition response, sucrose-induced lateral root growth enhancement, and primary root growth suppression, under nutrient starvation conditions, such as carbon and nitrogen limitation (Jeon et al., 2021).

*CEP5* negatively regulates primary and lateral root development (Roberts et al., 2016) and plays a role in osmotic and drought stress tolerance in Arabidopsis (Smith et al., 2020). Proteome analysis revealed that *CEP5* alters significant portions of the proteins involved in the biological processes “response to stress or to abiotic stimulus”, indicating a potential role of *CEP5* in abiotic stress responses. Consistent with this, *CEP5*-overexpression lines or wild type-seedlings treated with hydroxyprolinated *CEP5* peptide exhibit better recovery from drought after re-watering than wild type, indicating that *CEP5* can confer drought stress tolerance. *CEP5*-overexpression line also displays enhanced tolerance in rosette size reduction to osmotic stress and enhanced expression of osmotic stress-inducible transcription factor genes. The *xylem intermixed with phloem (xip)*/*cepr1 cepr2* double mutant did not display rosette size reduction upon osmotic stress treatment, indicating that CEP5 acts independently of the CEPRs. Notably, *CEP5*-overexpression line displays significant reduction in both *DR5-GUS* or *DR5-LUC* activities and expression of auxin-inducible genes, *LOB DOMAIN-CONTAINING PROTEIN 18* (*LBD18*), *LBD29*, and *PIN-FORMED 1* (*PIN1*). CEP5 stabilizes AUX/IAA proteins, negative regulators of AUXIN RESPONSE FACTORs, by affecting proteasome activity but without altering auxin levels and auxin transport activity (Figure 1D). Whether and how the negative regulatory role of CEP5 in auxin response through AUX/IAA stabilization is linked to drought and osmotic stress tolerance need further investigation. Functional analysis of the *cep5* mutants is also necessary to support the results obtained with *CEP5* overexpression.

## Salinity Stress Response

### CAPE Peptides

CAP-derived peptide 1 (CAPE1) is derived from the C-terminus of PATHOGENESIS-RELATED PROTEIN1b (PR-1b), a member of the cysteine-rich secretory proteins, antigen 5, and pathogenesis-related 1 proteins (CAP) superfamily (Chen et al., 2014). CAPE1 was initially identified using a peptidomics approach from tomato leaves (Chen et al., 2014). CAPE1 induces significant anti-pathogen response in tomato, indicating a role for PR-1 in immune signaling (Chen et al., 2014). In *Arabidopsis*, AtCAPE1, comprising 11 amino acids, negatively regulates salt-stress tolerance (Chien et al., 2015; Figure 1E). Nine potential CAPEs from *Arabidopsis* were identified as precursor candidates for CAPEs on the basis of sequence similarity to the precursor of tomato CAPE1 and the C-terminal conserved motif, and were named as the precursor *Arabidopsis thaliana* CAPEs (PROAtCAPEs) (Chien et al., 2015). Among them, *PROAtCAPE1* is down-regulated mainly by salt stress. The *proatcape1* mutant exhibits resistance to growth inhibition under high-salt conditions, whereas exogenous application of synthetic AtCAPE1 peptide or overexpression of *PROAtCAPE1* restores the sensitive phenotype of the mutant, indicating that AtCAPE1 functions as a negative regulator of salt-stress tolerance in *Arabidopsis*. AtCAPE1also negatively regulates salt-inducible genes, such as those involved in osmolyte biosynthesis, detoxification, and dehydration response (Chien et al., 2015). Hence, AtCAPE1 plays a role in the regulation of salt stress responses in *Arabidopsis*.

### RALF Peptides

Rapid alkalinization factor (RALF) peptides are 5 kDa cysteine-rich peptides inducing rapid alkalinization of the extracellular compartments of plant cells, thus reducing the proton electrochemical potential required for solute uptake and causing cell growth suppression (Blackburn et al., 2020). RALF peptides are involved in root growth, immune responses, guard cell movement, and pollen tube growth and termination during plant reproductive processes (Ge et al., 2017; Mecchia et al., 2017; Blackburn et al., 2020). They are recognized by the LRR-RLK FERONIA (FER) (Stegmann et al., 2017). RALF22/23-FER module regulates salt tolerance by interacting with the cell-wall LRR extensins (LRX)3/4/5 (Zhao et al., 2018; Figure 1E). LRX proteins comprise an N-terminal LRR and a C-terminal extension domain and are localized to the cell wall, playing a role in cell wall-plasma membrane communication (Fabrice et al., 2018). The *lrx3/4/5* triple mutant, *fer* mutant, and *RALF22* or *RALF23*-overexpressing plants all display similar phenotypes, such as reduced plant growth and salt hypersensitivity (Zhao et al., 2018). RALF peptides are physically associated with LRX and FER proteins (Zhao et al., 2018). Salt stress causes dissociation of mature RALF22 peptides from LRX proteins, thereby inducing FER internalization via an endosomal pathway (Zhao et al., 2018; Figure 1E). Therefore, RALF22/23-FER and LRX3/4/5 regulate plant growth and salt tolerance via salt-induced cell wall changes.

FERONIA is also necessary for cell-wall integrity, preventing root cells from bursting during growth under high salt stress (Feng et al., 2018). The glycosylphosphatidylinositol-anchored protein (GPI-AP) LORELEI-like GPI-AP1 (LLG1) interacts directly with FER in the endoplasmic reticulum and is required for both FER localization to the plasma membrane and FER-mediated RHO GTPase signaling (Li et al., 2015). *llg1* mutant roots display the same ionic sensitivity and cellular damage caused by a loss of cell-wall integrity as the *fer* mutant (Feng et al., 2018), indicating that FER-LLG1 interaction is important for maintaining cell-wall integrity in the roots. Fortification of pectin cross-links restores growth and cell-wall integrity of *fer* seedlings under salt stress (Feng et al., 2018). The FER extracellular domain binds pectin *in vitro*. FER is necessary for salinity-induced [Ca^2+^] transients to maintain cell wall integrity during growth recovery, suggesting that FER induces calcium signaling in response to salt stress to maintain cell wall integrity by physically interacting with pectin in the cell wall (Figure 1F). Further investigation is needed to identify the calcium channel responsible for FER-mediated induction of calcium transients and how such transients regulate downstream signaling to repair salt stress-induced cell-wall damage. The *ralf1* mutant and a *RALF1* RNAi transgenic line did not show significant root growth inhibition, indicating that RALF1 does not display a critical function in regulating root growth under salt stress (Feng et al., 2018). As RALF22/23-FER module regulates plant growth and salt tolerance via salt-induced cell-wall changes (Zhao et al., 2018), RALF22/23 might play a role in FER-mediated protection of root cells from bursting under salt stress.

### AtPEP3

Plant elicitor peptides (Peps) are endogenous elicitors of pattern-triggered immunity against bacteria, fungi, and herbivores (Yamaguchi and Huffaker, 2011; Bartels and Boller, 2015). Among eight members of the *Arabidopsis thaliana* precursor Pep (*AtPROPEP*) family in *Arabidopsis*, AtPep3 plays a role in salinity stress response (Nakaminami et al., 2018). Among the *AtPROPEP* gene family, *AtPROPEP3* displays the greatest induction in response to high salinity. Overexpression of *AtPROPEP3* or exogenous application of synthetic AtPep3 peptide (30 amino acids) derived from the C-terminal region of PROPEP3 induces salt stress tolerance (Nakaminami et al., 2018). Conversely, *AtPROPEP3*-RNAi lines are hypersensitive under salinity stress, which is recovered by AtPep3 peptide application. Endogenous AtPep3 peptide was further identified from NaCl-treated plants via mass spectrometry (Nakaminami et al., 2018). AtPEP1-6 peptides bind to the LRR-RLK PEP RECEPTOR1 (PEPR1) and AtPEP1/2 peptides bind to PEPR2 to initiate immune signaling (Yamaguchi et al., 2006, 2010). Although high-salt treatment significantly reduces plant survival of the *pepr1*, *pepr2*, and *pepr1/2* mutants, exogenous AtPep3 peptide application recovers plant survival only in the *pepr2* mutants (Nakaminami et al., 2018), indicating that AtPep3 is recognized by the PEPR1 receptor to induce salinity stress tolerance in plants (Figure 1E). Hence, AtPEP3–PEPR1 module may have a dual function in both salinity stress tolerance and immune responses.

## ROS-Mediated Stress Signaling

### IDA-LIKE (IDL) Peptides

Bioinformatic analyses identified eight *IDL* genes with similarity to IDA (Butenko et al., 2003; Vie et al., 2015). *In silico* analysis showed that *IDL6* and *IDL7* respond to biotic and abiotic stresses, such as cold, salt, and UV light in the root (Vie et al., 2015). Application of IDL6 and IDL7 peptides to *Arabidopsis* reduces the expression of early stress-responsive genes, including *ZINC FINGER PROTEIN* and *WRKY* genes (Vie et al., 2017). *IDL7* expression is rapidly induced by the ROS hydrogen peroxide, and ROS-induced cell death is mitigated in the *idl7* mutant. IDL7 peptides can attenuate the rapid ROS burst induced by the bacterial elicitor flagellin 22 (flg22), whereas *IDL7* mutation enhances flg22-induced ROS burst. Hence, IDL7 acts as a negative regulator of stress-induced ROS signaling in *Arabidopsis* (Figure 1G). As *IDL6* displays expression patterns similar to *IDL7* in response to ROS and IDL6 downregulates similar target genes, IDL6 might act like IDL7 in ROS signaling. Although HAE/HSL2 are likely to perceive IDL6 and IDL7 peptides, the receptors of IDL6/7 peptides remain to be experimentally identified (Vie et al., 2017).

## Heat Stress Response

### CLE45 Peptide

Environmental stresses negatively affect reproductive development of flowering plants and thus the seed yield (Giorno et al., 2013; De Storme and Geelen, 2014). To date, one signaling peptide is reported to play a role in high temperature stress response in plants (Endo et al., 2013), i.e., the *Arabidopsis CLE45*-STERILITY-REGULATING KINASE MEMBER1 (SKM1)/SMK2 receptor module in pollen tube growth (Endo et al., 2013). *CLE45* prolongs pollen tube growth in *in vitro* pollen tube culture. Further, the double mutation in *SKM1/2* encoding LRR–RLKs causes insensitive phenotype in pollen tube growth *in vitro* in response to synthetic CLE45 peptide. Photoaffinity labeling experiment with the SKM1-HaloTag protein demonstrated that SKM1 physically interacts with CLE45 peptides. GUS reporter expression analysis and artificial crossing experiments suggest that heat-inducible *CLE45* in pistils and *SKM1* in pollen function in the same signaling pathway. Suppression of *CLE45* expression by RNAi or expression of a kinase-dead version of SKM1 acting as a dominant negative form of SKM1 in the *skm1* mutant reduces seed number and size at 30°C but not at room temperature (22°C). Collectively, these findings suggest that the *CLE45*–SKM1/2 pathway sustains pollen tube growth under high temperatures, leading to successful seed production (Figure 1H).

## Nutrient Deficiency Stress Responses

**FIGURE 2:**
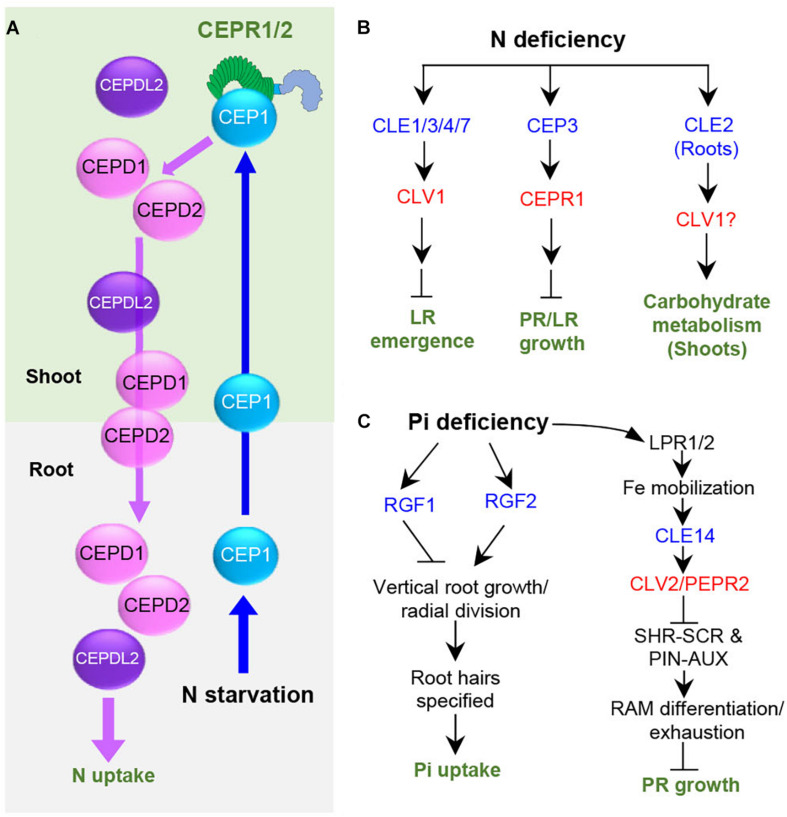
Role and signaling pathways of peptides regulating plant responses under nutritional deficiency stresses in Arabidopsis. Blue and red letters indicate peptides and the cognate receptors, respectively. **(A)** The systemic N-acquisition response. **(B)** Plant responses under nitrogen deficiency. **(C)** Plant responses under phosphate deficiency. N, nitrogen; Pi, inorganic phosphate; LPR, Low Phosphate Root2; LR, lateral root; PR, primary root.

Nitrogen (N) and inorganic phosphate (Pi) are the most important nutrients required by plants, and thus their deficiency profoundly influences the morphology, physiology, growth, and development in plants (Reddy, 2006). Peptides, such as CEPs, CLEs, and RGFs, were identified to play a role in the regulation of nutrient acquisition and to affect plant physiology and growth responses. CEP1 acts as a root-to-shoot mobile signal to promote compensatory N acquisition in the other N-rich parts of the roots (Okamoto et al., 2016). CEP1 peptides originating from the roots under N deficiency are translocated through the xylem to the shoots, where CEP1 peptides are recognized by XIP1/CEPR1 and CEPR2 receptors, generating descending shoot-to-root signals, CEP Downstream 1 (CEPD1) and CEPD2, which belong to the glutaredoxin family (Tabata et al., 2014; Ohkubo et al., 2017; Figure 2A). CEPDs are transported to the roots through the phloem to upregulate the nitrate transporter gene *NRT2.1* for promoting N uptake (Ohkubo et al., 2017). CEPD-like 2 originating from the shoots promotes both high-affinity N-uptake in the roots and root-to-shoot transport of nitrate, acting cooperatively with CEPD1/2 (Ota et al., 2020). CEP3 controls nutrient starvation response that suppresses root growth under N-limitation condition, potentially to enhance seedling survival (Delay et al., 2013, 2019; Figure 2B).

*CLE3* inhibits lateral root growth, likely through the receptor CLV1, as the *clv1* mutant displayed longer lateral roots under N deficiency and the inhibitory action of *CLE3* in lateral root formation was compromised in the *clv1* mutant (Araya et al., 2014; Figure 2B). *CLE1/3/4/7* genes are upregulated in root pericycle cells by systemic low N levels (Araya et al., 2014), implying that *CLE1/3/4/7* act in the combinatorial manner to control lateral root growth. *CLE2* expression is upregulated by N supply, dark, and sugar starvation (Ma et al., 2020). The *cle2* mutant plants exhibit a severe growth defect with leaf chlorosis under these conditions, whereas *cle3* did not display such growth phenotype under the same condition (Ma et al., 2020). Consistent with this phenotype, overexpression of *CLE2* in roots increased many different genes in shoots, such as dark-inducible genes and the genes involved in metabolite sensing and regulation of carbohydrate metabolism. These results indicate that *CLE2* induced in the roots functions systemically to control carbohydrate metabolism in light-dependent manner (Ma et al., 2020; Figure 2B). It is of interest to identify how a single amino acid difference between CLE2 and CLE3 peptides renders these two similar peptides to regulate different plant responses in different tissues. CLE14 mediates low-phosphate stress signal in roots to trigger RAM differentiation and exhaustion through CLV2/PEPR2 receptors via suppressing SCARECROW (SCR)/SHORT-ROOT (SHR) and PIN/auxin pathway, inhibiting primary root growth (Gutiérrez-Alanís et al., 2017; Figure 2C). This *CLE14* signaling pathway allows *Arabidopsis* plants to adapt to Pi availability around the roots.

Plants alter root development under Pi scarcity to maximize Pi acquisition. Genetic and microscopic expression analyses suggest that *ROOT GROWTH FACTOR1* (*RGF1*) and *RGF2* regulate different aspects of root development under Pi-deprivation conditions (Cederholm and Benfey, 2015). RGF2 promotes vertical root growth and radial divisions, whereas RGF1 inhibits radial divisions in the root meristem, specifying root hairs for facilitating Pi uptake (Cederholm and Benfey, 2015; Figure 2C). RGF1 promotes root meristem activity via a mitogen-activated protein kinase cascade by inducing *RGF1-INDUCIBLE TRANSCRIPTION FACTOR* (*RITF*), which enhances the stability of a master regulator of root stem cells, PLETHORA2, through ROS signaling (Fernandez et al., 2013; Ou et al., 2016; Shinohara et al., 2016; Song et al., 2016; Fernandez et al., 2020; Lu et al., 2020; Shao et al., 2020; Yamada et al., 2020). It remains to be investigated whether this signaling pathway is involved in RGF-regulated root development caused by Pi deprivation.

## Conclusion and Perspectives

Over recent years, several peptides and signaling pathways have been identified to play roles in coordinating plant responses to abiotic stresses. Although the *Arabidopsis* genome alone encodes several thousands of small coding genes (Takahashi et al., 2019), only few peptides have been reported to be involved in controlling plant abiotic stress responses. Moreover, there exist many questions regarding molecular mechanisms of peptide signaling during abiotic stress responses to be addressed. Functional and molecular understanding on RLKs and the coreceptors that interact with signaling peptides in response to abiotic stress are limited. Questions remain on how peptide precursors are processed into mature peptides, and mature peptides are secreted and transported to the target tissues in long-distance peptide signaling pathways. How peptide-receptor interactions lead to specific developmental controls under abiotic stress need investigation. How peptide-mediated signaling pathways confer plant tolerance to abiotic stresses is also unknown. *CLE25* induces active ABA accumulation in the leaves through long-distance signaling from the roots to the leaves in response to dehydration, and *CLE9* uses ABA signaling components to enhance drought stress tolerance. It will be of interest to investigate how signaling peptides are linked to conventional phytohormone pathways under abiotic stress for obtaining stress resistance. How changes in local concentrations of nutrients and abiotic stressors are sensed in plants for eliciting peptide signaling will be a challenging issue to be explored. As abiotic stresses accelerated by climate changes are a major threat to crop yields and food security, the identification of plant peptides and signaling pathways will provide new strategies to improve stress tolerance in crops for agricultural sustainability.

## Author Contributions

JiK and BJ wrote the draft manuscript. JuK conceived the review outline and wrote and edited the manuscript. All authors contributed to the article and approved the submitted version.

## Conflict of Interest

The authors declare that the research was conducted in the absence of any commercial or financial relationships that could be construed as a potential conflict of interest.
